# Mitogen-Activated Protein Kinases: Functions in Signal Transduction and Human Diseases

**DOI:** 10.3390/ijms20194844

**Published:** 2019-09-29

**Authors:** Ritva Tikkanen, David J. Nikolic-Paterson

**Affiliations:** 1 Institute of Biochemistry, Medical Faculty, Justus-Liebig University of Giessen, Friedrichstrasse 24, D-35392 Giessen, Germany; 2 Department of Nephrology, Monash Health and Monash University Centre for Inflammatory Diseases, 246 Clayton Road, Clayton, Victoria 3168, Australia; david.nikolic-paterson@monash.edu

Mitogen-activated protein kinases (MAPKs) are involved in signaling processes induced by various stimuli, such as growth factors, stress, or even autoantibodies. Sequential activation and phosphorylation of kinases upstream of the MAPKs result in activation of the MAPKs and phosphorylation of their downstream substrates that can be either nuclear (e.g., transcription factors) or cytoplasmic (e.g., proteins involved in cell migration). MAPKs comprise three functional families, namely, extracellularly regulated kinases (ERKs), c-Jun N-terminal kinases (JNKs) and p38. MAPK signaling thus provides a versatile signaling module that regulates cell proliferation, differentiation, homeostasis and even cell death. 

Individual proteins associated with the MAPK signaling cascades are regulated not only by phosphorylation but also by scaffolding proteins that can associate with several signaling proteins simultaneously and thus facilitate their regulation and interaction. Numerous scaffolding proteins are known to exist (for a review, see [1]). In this Special Issue, Dietel et al. summarize the role of one of these scaffolders, protein tyrosine phosphatase interacting protein 51 (PTPIP51), in the regulation of MAPK signaling [2]. The authors provide an overview about the function of this interesting protein in physiological signaling, but also in various diseases associated with dysregulation of MAPK signaling. 

While MAPK pathways have homeostatic roles, the pathological mechanisms underpinning many disease processes involve signaling via one or more of the p38, JNK or ERK pathways. This Special Issue provides new insights into how MAPK signaling contributes to specific pathological processes across a range of conditions, including disorders of lung development, type 2 diabetes, cancer, proliferative skin diseases, cardiovascular disease and neurological disease. 

Bronchopulmonary dysplasia (BPD) is a chronic lung disease of premature infants characterized by interrupted lung development. Supplemental oxygen is critical for preterm infants with respiratory failure. However, this hyperoxic environment contributes to BPD pathogenesis. Using a newborn mouse model, Menon et al. [3] found that hyperoxia induced a transient increase in endothelial ERK1/2 signaling during the period of lung vascularization at day 7—possibly as an adaptive response to mitigate hyperoxic injury—but this signaling was not sustained at day 14 when endothelial apoptosis and development of BPD occurred. This finding was replicated in cultured fetal human pulmonary artery endothelial cells where hyperoxia induced ERK-dependent proliferation.

Excessive fibroblast growth factor receptor (FGFR) signaling has been shown to be involved in human diseases such as cancer and skeletal disorders [4]. Thus, negative feedback regulation of FGFR signaling (e.g., by ERK and p38 MAPK) is required to ensure that this signaling cascade is precisely controlled. FGFR substrate 2 (FRS2) is an adapter protein that becomes Tyr-phosphorylated during FGFR signaling and feeds the signal forward towards MAPK activation. A negative feedback regulation loop that involves ERK-mediated Thr phosphorylation of FRS2 has been shown to be a major downregulation mode for FGFR signaling [5]. In this Special Issue, Zakrzewska et al. provide a deeper insight into this important feedback mechanism by showing that not only ERK, but also p38 kinase can also phosphorylate FRS2 and thus contributes to the negative regulation of FGFR [6]. This regulation mechanism appears to require the activity of both ERK and p38 kinases, since inhibition of both kinases resulted in prolonged FGFR signaling. 

Activation of MAPKs, in particular JNKs, has been shown to inhibit insulin signaling and contribute to inflammation in type 2 diabetes [7]. In this issue, Cui et al. [8] show that the traditional Chinese medicines *Scutellaria baicalensis* Georgi and *Coptis chinensis* Franch, individually and in combination gave significant protection against hyperglycemia, hyperinsulinemia and lipid abnormalities in a rat model of type 2 diabetes. This was associated with reduced systemic and liver inflammation, with data suggestive of reduced JNK, NF-κB, and Akt signaling as the underlying protective mechanisms. Interestingly, the effects of these traditional medicines are comparable to that of metformin that acts via AMP-activated protein kinase signaling which inhibits JNK signaling [7,8].

Activation of JNK signaling pathways is also associated with other pathological processes such as liver diseases. In this Special Issue, Win et al. discuss the implications of JNK signaling mode as a modulator of cell death [9]. The authors stress that not only the level of signaling activation, but also its duration is important for the signaling outcome. In their review article, Win et al. mainly focus on the interplay of JNK signaling with the mitochondrial SAB (SH3 domain binding protein 5) protein that promotes the release of reactive oxygen species (ROS) and cell death. Therefore, modulation of SAB function and thus JNK signaling may provide means for therapy in conditions such as cancer, in which the balance between survival and cell death frequently involves the JNK-SAB-ROS axis. 

In addition to MAPK, death-associated protein kinases (DAPKs) have been shown to be important regulators of cell death. In this Special Issue, Elbadawy et al. provide an overview of the functions of DAPKs in various diseases such as cancer, neuronal injury and cardiovascular disease [10]. A special focus of this review is the crosstalk of DAPK and MAPK signaling pathways and its implication for diseases, as well as the potential of DAPKs as therapeutic targets. As small molecules that inhibit DAPKs have become available, it will be interesting to see if these can be used for the treatment of the diseases associated with DAPK activity. 

Infantile myofibromatosis is a rare disorder of mesenchymal proliferation characterized by nonmetastatic tumors and which is associated with specific point mutations in the platelet-derived growth factor receptor B (PDGFRB) gene. Using primary tumor samples and the tumor-derived NSTS-47 cell line, Sramek et al. [11] demonstrated that a PDGFRB mutation results in prominent PDGFRB/ERK signaling. Interestingly, while tumor cell proliferation was resistant to ERK inhibitors, the non-selective tyrosine kinase inhibitor sunitinib inhibited tumor cell proliferation via reducing PDGFRB and Akt phosphorylation, explaining the beneficial effects seen with this drug in some patients [12].

Another chronic proliferative disease is psoriasis, a multisystemic disease characterized by abnormal keratinocyte proliferation resulting in erythematous lesions on the skin. Dermal fibroblasts can regulate the function of adjacent keratinocytes, and studies by Becatti et al. [13] identified a marked activation of p38 and JNK signaling pathways in fibroblasts from psoriatic lesions versus control fibroblasts. This enhanced kinase signaling was associated with increased mitochondrial superoxide production and apoptosis of psoriatic fibroblasts. Mechanistically, psoriatic fibroblasts exhibited reduced expression of the NAD^+^-dependent protein deacetylase, SIRT1. Addition of the SIRT1 activator, SRT1720, diminished p38 and JNK signaling and normalized function in psoriatic fibroblasts, suggesting therapeutic potential for this approach. 

Inflammatory bowel disease (IBD) is characterized by intestinal barrier dysfunction that is strongly associated with gastrointestinal infections caused by Gram-negative bacteria. Cecropin A is an antimicrobial peptide (AMP) that has been shown to exhibit antimicrobial activity, but its potential role in intestinal barrier function has remained unknown. In this Special Issue, Zhai et al. now show that cecropin A can increase the expression of tight junction proteins zonula occludens 1 (ZO-1), claudin 1 and occludin, thus increasing intestinal cell barrier function [14]. Cecropin A treatment also resulted in downregulation of ERK phosphorylation, and chemical inhibition of ERK activity showed a synergistic effect on barrier function when applied together with cecropin A. These data are highly interesting for the treatment of IBD, since resistance to antibiotics that are used for the treatment of IBD-associated bacterial infections is a major problem. Therefore, AMPs such as cecropin A may provide an alternative as antibacterial therapy in IBD or other diseases associated with compromised intestinal barrier function. 

MAPK signaling is also involved in other gastrointestinal inflammatory diseases such as gastric ulcer. In this issue, Akanda et al. show that an extract from the perennial shrub *Rabdosia inflexa* (RI) exhibits potent anti-inflammatory and gastro-protective effects by affecting proinflammatory cytokine release and MAPK signaling [15]. RI was able to protect RAW 264.7 macrophage cells against various toxic stimuli such as lipopolysaccharide, NO and ROS. The authors showed that RI treatment resulted in reduced inflammatory signs, such as cyclooxygenase 2 expression or nuclear factor kappa B (NF-κB) activation. Importantly, gastric damage in HCl- and ethanol-treated mice was efficiently reduced by RI. Thus, RI, which is traditionally used in Chinese medicine for gastrointestinal problems, may exert its beneficial effects against inflammatory diseases of the gut by regulating MAPK/NF-κB signaling and cytokine release.

Atherosclerosis and aortic valve sclerosis are major cardiovascular diseases in Western societies. Reustle and Torzewski [16] discuss how p38 MAPK signaling in endothelial cells, vascular smooth muscle cells and (myo)fibroblasts may contribute to the pathogenesis of these conditions and make the argument that p38 MAPK inhibition may be useful in the treatment of these diseases. 

Bones are subject to continuous remodeling that involves osteoclasts that degrade the bone and the counteracting osteoblasts that reform the bone. Physiologically, it is important that the activity of these cells is kept in a delicate balance, and tipping of this balance in one or the other direction is involved in numerous diseases. In this issue, Lee et al. review the roles of MAPKs in osteoclast biology [17]. Osteoclast differentiation and activation are regulated by MAPK signaling, including ERK, JNK and p38. In their review, Lee et al. discuss the upstream regulation of MAPK signaling in osteoclasts and provide insights into the differential kinetics and crosstalk of the three MAPK signaling modes during osteoclast function.

ERK5, an atypical member of the ERK family, is thought to contribute to neuron differentiation and survival. In this context, Kashino et al. [18] used the PC-12 cell line to describe a mechanism whereby ERK5 phosphorylates the cytoplasmic domain of the Kv4.2 voltage-gated K^+^ channel to inhibit the A-type current inactivation, leading to rapid repolarization towards resting potential, thus causing an increase in firing frequency, which may contribute to the neuronal differentiation process. 

Parkinson’s disease (PD) is a neurodegenerative disorder caused by insufficient dopamine production due to the loss of dopaminergic neurons. Both oxidative stress and endoplasmic reticulum stress are directly implicated in the pathogenesis of PD. As reviewed by Bohush et al. [19], both of these stressors are potent activators of JNK and p38 MAPK signaling which induce both apoptosis of neurons and microglial activation, leading to chronic neuroinflammation, identifying these kinases as potential therapeutic targets in this disease. 

Organometallic drugs such as naphthalimides and their derivatives have shown promise in the treatment of cancers, but their clinical use is limited by their toxic side effects [20]. Thus, there is great interest in developing less toxic and efficient anticancer agents based on organometallic derivatives. In this issue, Dabiri et al. characterize the molecular mechanisms of rhodium(I) and ruthenium(II) containing naphthalimides conjugated with an *N*-heterocyclic carbene (NHC) moiety [21]. They show that these compounds induce a profound activation of p38 MAPK and elevated generation of reactive oxygen species, without affecting other MAPK signaling modules. These findings thus suggest that the effect of organometallic naphthalimides may be exerted through p38 signaling cascade. 

There have been numerous setbacks in the clinical use of MAPK inhibitors, in particular due to acute liver toxicity with p38 inhibitors [22]. However, there is renewed interest in targeting MAPKs in disease. Recently, the ERK1/2 pathway inhibitor trametinib was granted an accelerated approval by the US Food and Drug Administration for the treatment of unresectable metastatic melanoma. Apoptosis signal-regulating kinase 1 (ASK1/MAP3K5) is an upstream activator of p38 and JNK pathways, and an ASK1 inhibitor, selonsertib, is currently in phase 3 trials in liver fibrosis and diabetic kidney disease. Finally, CC90001 is a JNK inhibitor currently in phase 2 trials of idiopathic pulmonary fibrosis and in liver fibrosis. Thus, there is reason for optimism that targeting the pathological role of MAPK signaling may provide therapeutic benefit across a range of human diseases. 
